# Magnetic nanoantioxidants with improved radical-trapping stoichiometry as stabilizers for inhibition of peroxide formation in ethereal solvents

**DOI:** 10.1038/s41598-019-53531-5

**Published:** 2019-11-20

**Authors:** Caterina Viglianisi, Alessia Scarlini, Lorenzo Tofani, Stefano Menichetti, Andrea Baschieri, Riccardo Amorati

**Affiliations:** 1 Department of Chemistry “U. Schiff”, University of Florence, Via della Lastruccia 3-13, 50019 Sesto Fiorentino, Firenze Italy; 2 Department of Chemistry “G. Ciamician”, University of Bologna, Via S. Giacomo 11, 40126 Bologna, Italy

**Keywords:** Chemical safety, Nanoparticle synthesis

## Abstract

Graphite-coated magnetic cobalt nanoparticles (CoNPs) decorated with hindered phenolic antioxidant analogues of 2,6-di-*tert*-butyl-4-methylphenol (BHT, E321) provided easily removable nanoantioxidants capable of preventing the autoxidation of organic solvents as tetrahydrofuran (THF).

## Introduction

Tetrahydrofuran (THF) is one of the most popular ethereal solvents and together with ethyl ether, cyclopentyl methyl ether and 2-methyltetrahydrofuran, forms peroxides upon storage in the presence of O_2_ via an autoxidation mechanism^1^. Peroxides are highly unstable and represent an explosion hazard at concentrations above 100 ppm^2–5^. Autoxidation is a radical chain reaction sustained by peroxyl radicals (ROO^●^) which causes the oxidative degradation of organic compounds under mild conditions, leading to the formation of a variety of unwanted and in some case toxic and/or dangerous compounds including peroxides and epoxides^1,6^. Chain-breaking antioxidants (AH) counteract autoxidation by quenching the chain-carrying peroxyl radicals (Eq. 1–2)^7^.

1 $${\rm{ROO}}\bullet +{\rm{AH}}\to {\rm{ROOH}}+{\rm{A}}\bullet $$

2 $${\rm{ROO}}\bullet +{\rm{A}}\bullet \to {\rm{non}} \mbox{-} {\rm{radical}}\,{\rm{products}}$$

The hindered phenol butylated hydroxytoluene (BHT, 2,6-di-tert-butyl-4-methylphenol, E 321) is the stabilizer of choice for ethereal solvents, to which it is typically added in 100–300 ppm concentrations^8^. However, there are some limitations in the use of BHT, for example, it is incompatible with methods requiring high optical purity due to its UV absorbance, and must be avoided with solvent purification systems (such as Pure Solv^TM^ Micro or similar apparatuses) that permit the dispensing of small quantities of dry solvent^9^. In fact, BHT interacts with the filter material clogging the system, that allows only inhibitor-free solvents. Thus, to avoid dangerous peroxides formation, THF and other ethereal solvents have to be purchased frequently and in small quantities, limiting the effectiveness of these apparatuses. Indeed, an ideal solvent stabilizer should be easily and quantitatively removed just before use.

**Figure 1:**
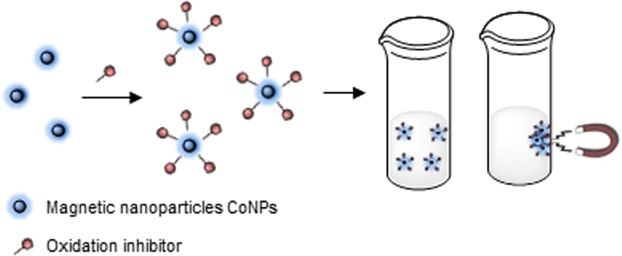
Schematic preparation, use and removal of nano-magnetic stabilizer systems.

We imagined that this problem could be solved by using oxidation inhibitors linked to magnetic nanoparticles that allows their removing by decantation using a magnet (Fig. 1).

The possibility to use nanomaterials as radical trapping agents represents a relatively unexplored frontier of the development of novel nano-supported antioxidants. Their small dimensions endow unique properties, which can be used to improve the performances of antioxidants, and to obtain hybrid materials that have been named “nanoantioxidants”^10^. Some relevant examples include the superoxide dismutase activity of several metal and metal-oxide nanoparticles^11,12^, the covalent link of gallic acid to the surface of silica nanoparticles to obtain a non-migrating antioxidant^10^, and the combined inclusion and surface linkage of antioxidants to halloysite nanotubes (such as diphenylamine derivative for polymer stabilization)^13–15^. We have previously reported the preparation of soft ferromagnetic cobalt nanoparticles (CoNPs) decorated on the surface with Trolox (a water-soluble analogue of vitamin E)^16^.

A problem affecting the efficiency of nanoantioxidants is that the number of radicals trapped by phenols linked to the surface of nanomaterials is usually smaller than 2, that is the value expected from reactions 1 and 2, because of the occurrence of dimerization between the surface-bound phenoxyl radicals. Herein, we describe the synthesis of novel magnetic nanoantioxidants opportunely designed to increase the efficiency of radical trapping by the insertion of bulky groups in *ortho* position to the reactive OH group, and their use for the stabilization of tetrahydrofuran from oxidative degradation.

## Results and Discussion

As magnetic nanoparticles, we choose commercially available soft ferromagnetic CoNPs of about 30 nm of diameter functionalized on the surface with azido groups (Turbobeads Click, CoNPs-N_3_) with a functional group-loading as 0.1 mmol/g^17–19^, that we previously reported to be suitable for the development of antioxidant systems. In fact, the metallic core is coated with roughly three graphitic layers that increases its stability and avoids pro-oxidant effects, as reported, for instance, for iron oxide magnetic nanoparticles^20,21^. The presence of azido groups allows the functionalization by a copper (I) catalysed azide-alkyne cycloaddition (CuAAC)^22^ to obtain CoNPs-Antiox. As pendants, we chose BHT-like molecules because steric crowding around its phenoxyl group is expected to prevent the stoichiometry reduction (number of radicals trapped by each molecule) due to radical-radical coupling, and because BHT is the molecule of choice for solvents and polymers stabilization^23–27^.

**Figure 2:**
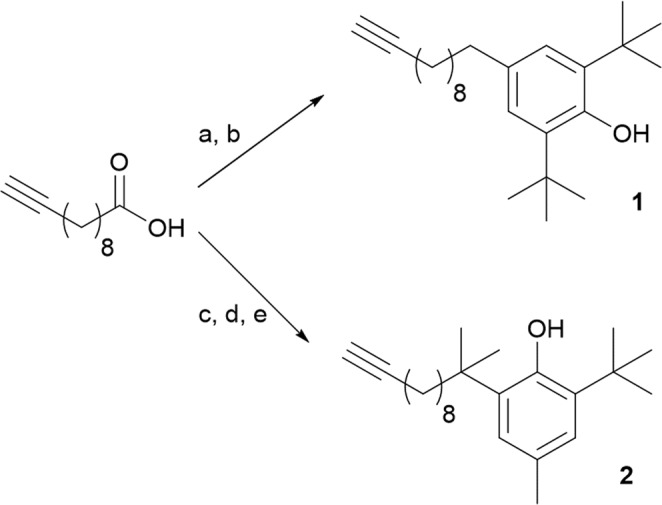
Synthesis of BHT-like derivatives **1** and **2** suitable for the functionalization of CoNPs-N_3_. (**a**) 2,6-di-*tert*-butylphenol, (CF_3_CO)_2_O, rt, 3 h; (**b**) activate Zn, HCl, CH_3_COOH, EtOH, reflux, 15 h; (**c**) SOCl_2_, MeOH, rt, 1 h; (**d**) MeMgBr, Et_2_O, rt, 16 h; (**e**) 2-*tert*-butyl-4-methylphenol, CH_3_SO_3_H, 1,2-DCE, rt, 24 h.

Two different BHT-like pendants were prepared, depending on the position of the linkage respect the reactive phenolic group (compounds **1** and **2**) (Fig. 2). The BHT moiety was separated from the alkyne functional group, needed for the CuAAC reaction, by a chain of nine carbon atoms. Commercially available 10-undecyn-1-ol was oxidized to 10-undecynoic acid^28^ which, in a one-pot procedure, through a Friedel-Crafts acylation with 2,6-di-*tert*-butylphenol catalysed by trifluoroacetic anhydride followed by a Clemmensen reduction promoted by activated zinc, concentrated HCl and acetic acid, allowed the isolation of BHT-like derivative **1** in 44% overall yield.

The second BHT-like alkyne derivative **2**, suitable for the functionalization of CoNPs-N_3_, was prepared from 10-undecynoic acid, used as common starting material, that was esterified with SOCl_2_ and methanol^29,30^ and then reacted with an excess of methyl magnesium bromide to give the corresponding tertiary alcohol^31^. After a non-trivial optimization of the reaction conditions, the Friedel-Crafts alkylation of 2-tert-butyl-4-methylphenol with this tertiary alcohol, using CH_3_SO_3_H as catalyst, allowed the isolation of alkyne **2** in 27% overall yield (Fig. 2).

**Figure 3:**
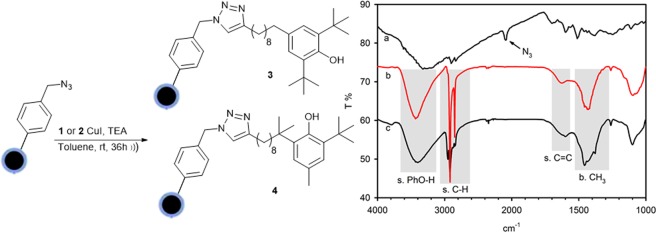
Functionalization of CoNPs-N_3_ under CuAAC conditions; Left: FT-IR (KBr pellets) spectrum of CoNPs-N_3_ (curve a) and FT-IR spectra of CoNPs-A80 **3** (curve c) and CoNPs-A94 **4** (curve b).

The derivatives **1** and **2** (10 equiv) were reacted with CoNPs-N_3_ (1 equiv) using CuI (0.5 equiv) in the presence of triethylamine (TEA, 5 equiv) as base and anhydrous toluene as solvent (Fig. 3). The suspension was sonicated [)))] at room temperature under nitrogen for 24 h. After a second addition of the same amount of CuI, the sonication was coninued for further 12 h. The use of an excess of alkyne and a “stoichiometric” amount of the “catalytic” copper salt is mandatory since to have the complete functionalization of the surface azido groups as pointed out by FT-IR (vide infra). The nanoparticles were recovered from the reaction mixture with the aid of a neodymium-based magnet and washed with toluene (2 × 9 mL). From this solution alkynes **1** and **2**, used in excess, were recovered and recycled. Trace of residual Cu(I) salt “trapped” on CoNPS surface were removed by washing with a solution of 33% NH_3_/EtOH (2/1, 5 mL) under sonication for 30 min. Then the solution was removed, and the nanoparticles washed with, in sequence, water (2 × 4 mL), EtOH (2 × 4 mL) and DCM (2 × 4 mL). Each washing step consisted of suspending the particles in the solvent, sonication (5 min) and retracting the particles from the solvent by the aid of the magnet. The functionalization of CoNPs-N_3_ to CoNPs-A80 (**3**) and CoNPs-A94 (**4**) could be easily detected by FT-IR (KBr pellets). From the complete disappearance of the N_3_ stretching peak at 2090 cm^−1^ we considered that the yield of the functionalization was 100% and relying on manufacturer data to assess the particle loading (0.1 mmol/g) it was possible to calculate the corresponding amount of antioxidant moieties (Fig. 3)^32^.

**Figure 4:**
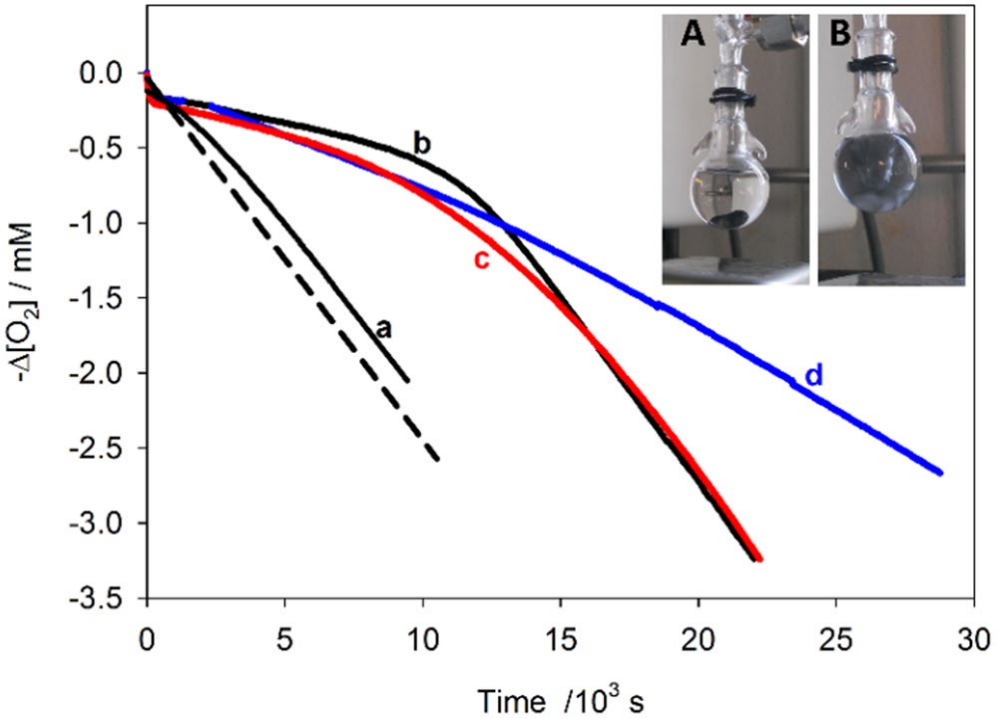
Oxygen consumption measured during the autoxidation of cumene (4.3 M) initiated by AIBN (25 mM, rate of initiation (*R*_*i*_) (2.2 ± 0.1) × 10^−9^ M^−1^ s^−1^ at 30 °C in benzonitrile without antioxidants (dashed line) or in the presence of (**a**) CoNPs-N_3_ (0.18 mg/mL); (**b**) BHT (18 *μ*M), (**c**) CoNPs-A94 **4** and (**d**) CoNPs-A80 **3** (both 0.18 mg/mL, corresponding to 18 *μ*M of linked BHT units). Magnetic nanoparticles adhered to the stir bar (inset A) but were homogeneously dispersed in solution when stirring was switched on (inset B).

The antioxidant activity of the CoNPs-A80 (**3**) and CoNPs-A94 (**4**) was assessed by measuring the inhibition of the autoxidation of cumene (isopropylbenzene), a reference substrate for autoxidation studies, initiated by azobis(isobutyronitrile) (AIBN) at 30 °C in benzonitrile (see Scheme S1 in the ESM)^33^. The reaction was followed by using an automatic gas absorption apparatus, based on a pressure transducer, built in our laboratory^33^. Figure 4 show the typical oxygen consumption traces recorded during the autoxidation of cumene in the presence of BHT and of the CoNPs-Antiox **3** and **4** in benzonitrile. CoNPs-Antiox generated an inhibition period, which was similar to that caused by BHT when used at a concentration corresponding to the molarity of the active phenolic moiety present on the nanoparticles, considering the quantitative functionalization of all the pending azide groups (see Fig. 4). By using a well assessed kinetic treatment (see ESM)^33^, from the O_2_ consumption traces the rate constants for the reaction with peroxyl radicals were determined as (7.0 ± 0.6) × 10^3^ M^−1^*s*^−1^ for BHT, in good agreement with the literature value considering the kinetic solvent effect^34,35^, and (4.0 ± 0.5) × 10^3^ or (2.6 ± 0.5) × 10^3^ M^−1^*s*^−1^ for the BHT moieties present on CoNPs-A80 **3** and CoNPs-A94 **4**, respectively.

The slightly lower k_*inh*_ value of CoNPs-Antiox **3** and **4** is likely due to steric shielding experienced on the surface of the nanoparticle. The stoichiometry of radical trapping (n) was determined as 2.2 ± 0.2 for BHT and 2.3 ± 0.2 or ≥4.2 for each BHT moiety present on CoNPs-A94 **4** and CoNPs-A80 **3**, respectively. The unexpected large n value of **3** may derive from the occurrence of secondary radical trapping mechanisms involving the formation of quinone methides and regeneration of the phenolic moiety by solvent addition^36^. These results can be compared to those obtained with unhindered phenols (such as Trolox^14,16^ or curcumin^15^) covalently linked to the surfaces of nanomaterials, as they were found to trap about one ROO^●^ radical per molecule of antioxidant. This remarkable difference enlightens the role of the bulky *tert* butyl groups in stabilizing the phenoxyl radicals and ensuring a good antioxidant effect to surface-bound phenols.

The efficacy of **3** (CoNps-A80) and **4** (CoNPs-A94) as stabilizers for ethereal solvents was evaluated by monitoring the development of hydroperoxides in THF by using a commercially available colorimetric assay (see Section 1 in the ESM).

**Figure 5:**
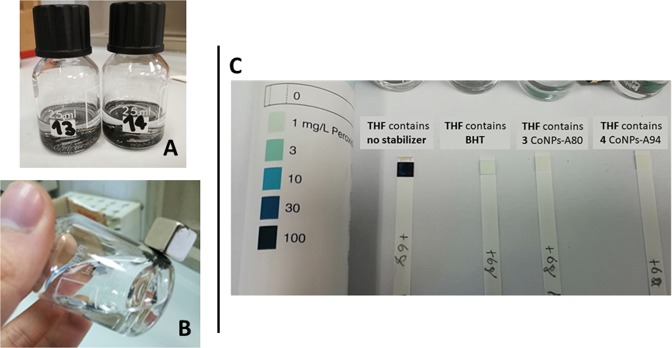
CoNPs-Antiox **3** and **4** suspended in THF (inset A) and recovered with a neodymium magnet (inset B), peroxide test strips (QUANTOFIX Peroxide 100, inset C).

In the presence of hydroperoxides in concentration higher than 1 mg/L, the colour of the test strip turns from white to blue and a quantification can be done by comparison to a colour scale (Fig. 5 inset C). The determination of peroxides formation in THF was done in a series of THF samples containing BHT, CoNPs-Antiox **3** and **4** all at 250 ppm (referred to the phenolic moieties) stored in clear glass vials at ambient light under continuous stirring. These conditions have been chosen to accelerate the oxidation and reduce the times of investigation. Actually, we proved that without continuous mixing the rate of peroxides formation increases sensibly. As shown in Fig. 5 inset C, after six day the THF sample without stabilizer reached a concentration of peroxides bigger than 100 mg/L as evident from the stick turning dark blue, the samples with BHT is about 1 mg/L, while those with CoNPs-A80 **3** and CoNPs-A94 **4**, inhibited the formation of peroxides, experimental details and pictures of test strips at different times are available in the ESM, see Fig. S3). The semi-quantitative strips peroxides tester does not allow to point out difference between the two nanoantioxidants materials **3** and **4**. However in Fig. 4 is shown the ability of both **3** and **4** in inhibiting peroxide formation measured in comparation with BHT. The quantitative removal of the nanomagnetic stabilizers could be easily accomplished by decantation using a neodymium magnet (Fig. 5 insets A and B). The weight of recovered CoNPs, after carefully drying with a N_2_ stream, was identical to that added ±5%.

In conclusion, magnetic nanoparticles CoNPs-Antiox **3** and **4** behave as good antioxidants able to inhibit the oxidative degradation of THF, an ethereal solvent of widespread use, with the possibility to simply remove the inhibitor with the aid of a magnet. The presence of bulky substituents *ortho* to the reactive OH group allowed to obtain a radical trapping stoichiometry superior to that of other phenols bound to nanomaterials. This approach avoids time and energy consuming procedures to remove stabilizers from easily oxidizable solvents or the use of potentially dangerous inhibitors-free solvents. Although, costs of CoNPs stabilizers reported are much higher than BHT, the problems related with the use of ethereal solvents without stabilizers in automatic drying dispensers may indicate a niche market for these materials. Indeed, we have preliminary similar result on the inhibition of peroxide formation in diethyl ether. Future work will aim to explore the possibility to improve the dispersion in organic solvents of different polarity and to obtain nanomagntic stabilizers able to prevent non-oxidative decompositions.

## Methods

### General information

^1^H- and ^13^C-NMR spectra were recorded with Varian Gemini 200 or Varian Mercury Plus 400. FT-IR spectra were recorded with FT Infrared Spectrometer 1600 Perkin-Elmer in CDCl_3_ solutions or KBr. GC-MS spectra were recorded with a QMD 100 Carlo Erba. ESI-MS spectra were recorded with a JEOL MStation JMS700. Melting points were measured with Stuart SMP50 Automatic Melting point. All the reactions were monitored by TLC on commercially available precoated plates (silica gel 60 F 254) and the products were visualized with acidic vanillin solution. Silica gel 60 (230–400 mesh) was used for column chromatography. Commercially available reagents and catalysts were used as obtained from freshly open container without further purifications. Dry solvents were obtained by Pure Solv^TM^ Micro Solvent Purification Systems. CHCl_3_ was washed 10 times with deionized water, dried on anhydrous CaCl_2_. Et_3_N was distilled over KOH. Cobalt nanoparticles were purchased from Turbobeads Llc; TurboBeads Click (CoNPs-N_3_) are carbon coated ferromagnetic cobalt nanoparticles (diameter 30 nm) which have a covalent azide functionality (0.1 mmol/g). Reactions with CoNPs were carried out under sonication with an ultrasonic bath (Sonorex RK 255 H-R, Bandelin). All air and moisture sensitive reactions were carried out in oven-dried glassware under a nitrogen atmosphere using cannulas and septa. Solvents were dried following standard procedures. Commercial zinc dust (Ø < 10 *μ*m) was activated by stirring for 4 min with 2% HCl. The zinc was immediately filtered in vacuo, washed to neutrality with water, and then washed with ethanol, acetone, and Et_2_O. The resulting power was dried at 90 °C under vacuum for 10 min and immediately used.

### Synthesis of new compounds

#### Undec-10-ynoic acid

To a solution 0.05 M of 10-undecyn-1-ol (971 mg, 5.77 mmol) in acetone (160 mL), Na_2_Cr_2_O_7_x2H_2_O (4304 mg, 14.44 mmol) and deionized water (5 mL) were added at 0 °C. Then conc. H_2_SO_4_ (1.5 mL) was added dropwise at 0 °C and the reaction mixture was left under magnetic stirring for 15 min. and filtered. The filtrate was extracted with AcOEt (3 × 30 mL) and the organic phase washed with water (3 × 30 mL) and brine (40 mL), dried over Na_2_SO_4_ and concentrated in vacuo. The desired product was obtained as a white solid without any further purification (1000 mg, 94% yield). ^1^H-NMR (CDCl_3_, 200 MHz) *δ* 1.26–1.67 (m, 12 H), 1.94 (t, *J* = 2.6 Hz, 1 H), 2.18 (dt, *J* = 6.9 Hz, *J* = 2.6 Hz, 2 H), 2.35 (t, *J* = 7.4 Hz, 2 H). **2,6-Di-tert-butyl-4-undec-10-ynyl-phenol (1)**. Undec-10-ynoic acid (484 mg, 2.66 mmol) was dissolved in 440 *μ*L of trifluoroacetic anhydride, then after 10 min 2,6-di-*tert*-butylphenol (413 mg, 2.00 mmol) was added at 0 °C. The so formed brown solution was stirred at 0 °C for 30 min. and then at room temperature until the disappearance of the starting phenol after 3 h (eluent for TLC control: Ep/Et_2_O 10/1). To the resulting dark solution absolute EtOH (10 mL), glacial acetic acid (5 mL) and 37% HCl (3.0 mL) were added, then the mixture was heated to reflux and activated Zn dust (<10 *μ*m, 3000 mg, 46 mmol) was added in small portions. The resulting colourless suspension was vigorously stirred under reflux for 18 h, then after TLC control (eluent: Ep/Et_2_O 15/1) the absence of the intermediate ketone was demonstrated, and the reaction was quenched with saturated aq. NaHCO_3_. The residual zinc was filtered off and the filtrate was extracted with petroleum ether (3 × 15 mL), then the organic phase was washed with saturated aq. NaHCO_3_ (3 × 20 mL) and water (3 × 20 mL). The organic phase was dried over anhydrous Na_2_SO_4_ and evaporated in vacuum furnishing a yellow oil that was purified by silica gel column chromatography (petroleum ether/DCM 10/1), giving desired product as a colourless oil of (306 mg, 44% yield). ^1^H-NMR (CDCl_3_, 400 MHz) *δ* 1.31–1.60 (m, 32 H), 1.95 (t, *J* = 2.6 Hz, 1 H), 2.19 (dt, *J* = 7.2 Hz, *J* = 2.6 Hz 2 H), 2.51 (t, *J* = 8.0 Hz, 2 H), 5.04 (s, 1 H), 6.98 (s, 2 H). ^13^C-NMR (CDCl_3_, 100 MHz) *δ* 18.4, 28.5, 28.7, 29.1, 29.5, 29.6, 30.3, 32.0, 34.2, 36.0, 68.0, 84.8, 124.8, 133.47, 135.50, 151.6. IR (CDCl_3_, cm^−1^) *ν* 3643, 3308, 2931, 2857, 2116, 1458, 1435. MS (ESI) m/z 355.58 [M-H]^-^. **Undec-10-ynoic acid methyl ester**. Thionyl chloride (600 *μ*L, 8.24 mmol) was added at −15 °C to a solution of undec-10-ynoic acid (750 mg, 4.12 mmol) in anhydrous MeOH (21 mL). The reaction mixture was left under magnetic stirring at room temperature for 1 h, then the solvent was evaporated. After the dilution with water, the mixture was extracted with Et_2_O (4 × 20 mL), washed with water until pH = 7 and finally with brine. The organic phase was dried over Na_2_SO_4_ and concentrated in vacuo, affording the desired ester as a yellow oil without any further purification (701 mg, 87%). ^1^H-NMR (CDCl_3_, 200 MHz) *δ* 1.31–1.67 (m, 12 H), 1.93 (t, *J* = 2.6 Hz, 1 H), 2.18 (dt, *J* = 6.9 Hz, *J* = 2.6 Hz, 2 H), 2.30 (t, *J* = 7.4 Hz, 2 H), 3.66 (s, 3 H). **2-Methyl-dodec-11-yn-2-ol**. In a Schlenk tube, to a solution of undec-10-ynoic acid methyl ester (1009 mg, 5.15 mmol) in anhydrous Et_2_O (20 mL), methylmagnesium bromide (3.0 M in Et_2_O, 8 mL, 26.00 mmol) was added dropwise at -78 °C. The mixture was left under magnetic stirring and N_2_ atmosphere allowing warming to room temperature, then after 15 h it was quenched with ice and acidified to pH 4 with aq. HCl 3 N. The so-obtained suspension was extracted with AcOEt (3 × 20 mL), then collected organic phases were washed with water (3 × 10 mL), dried over anhydrous Na_2_SO_4_ and evaporated in vacuum, furnishing a crude that was purified through flash column chromatography (petroleum ether/AcOEt 5/1) affording the desired product as a colourless oil (973 mg, 96%). ^1 ^H-NMR (CDCl_3_, 400 MHz) *δ* 1.20 (s, 6 H), 1.29–1.53 (m, 14 H), 1.93 (t, J = 2.6 Hz, 1 H), 2.17 (dt, *J* = 7.2 Hz, *J* = 2.6 Hz, 2 H). ^13^C-NMR (CDCl_3_, 100 MHz) *δ* 18.4, 24.3, 28.4, 28.7, 29.0, 29.2, 29.5, 30.1, 44.0, 68.1, 71.0, 84.8. IR (CDCl_3_, cm^−1^) *ν* 3607, 3308, 2934, 2858, 2116, 1467. **2-*tert*-Butyl-6-(1,1-dimethyl-undec-10-ynyl)-4-methylphenol (2)**. A solution of 2-methyl-dodec-11-yn-2-ol (200 mg, 1.02 mmol) and 2-*tert*-butyl-4-methylphenol (170 mg, 1.04 mmol) in 1,2-DCE (6 mL) was added dropwise at 0 °C to a suspension of CH_3_SO_3_H (439 mg, 4.57 mmol) in 1,2-DCE (1 mL). The resulting red solution was left under magnetic stirring at room temperature, monitored by TLC (eluent: petroleum ether/Et_2_O 4/1 and petroleum ether). After 24 h the reaction was quenched with saturated aq. NaHCO_3_, then the resulting mixture was diluted with DCM (30 mL) and washed with saturated aq. NaHCO_3_ (3 × 10 mL) and water (3 × 10 mL). The organic phase was dried over anhydrous Na_2_SO_4_ and evaporated in vacuo furnishing a yellow oil that was purified by silica gel column chromatography (petroleum ether fallowed by petroleum ether/DCM 10/1), giving the desired product **2** as a yellow oil (94 mg, 27% yield). ^1^H-NMR (CDCl_3_, 400 MHz) *δ* 1.04–1.39 (m, 10 H), 1.40 (s, 6 H), 1.43 (s, 9 H), 1.46–1.54 (m, 2 H), 1.77–1.81 (m, 2 H), 1.94 (t, *J* = 2.6 Hz, 1 H), 2.17 (dt, *J* = 7.0 Hz, *J* = 2.6 Hz, 2 H), 2.28 (s, 3 H), 5.02 (s, 1 H), 6.91 (s, 1 H), 6.99 (s, 1 H). ^13^C-NMR (CDCl_3_, 100 MHz) *δ* 18.40, 18.43, 21.3, 25.1, 28.5 28.7, 29.0, 29.19, 29.24, 30.2, 30.3, 34.2 37.7, 41.8, 68.1, 84.8, 125.5, 126.6, 128.0, 134.1, 135.5, 151.5. IR (CDCl_3_, cm^−1^) *ν* 3632, 3308, 2931, 2857, 1434, 1386. MS (ESI) m/z 283.50 [M-58]-, 341.58 [M-H]^-^. **CoNPs-A80 (3)**. Turbobeads Click (100 mg, 0.1 mmol/g azide-loading, 0.01 mmol) were washed with degassed toluene (3 × 1 mL) and suspended in the same solvent (4 mL) by sonication (10 min) before **1** (33 mg, 0.1 mmol,) TEA (7 *μ*L, 0.05 mmol) and CuI (1 mg, 0.005 mmol) were added. The resulting slurry was sonicated for 24 h at room temperature under a nitrogen atmosphere, then a second crop of CuI (1 mg, 0.005 mmol) was added and the mixture sonicated for additional 12 h. The nanoparticles were recovered from the reaction mixture with the aid of a neodymium-based magnet and washed with toluene (2 × 9 mL). Adventitious trace of residual Cu(I) salts were removed by washing with a solution of 33% ammonia/EtOH (2/1, 5 mL) under sonication for 30 min, then the solution was removed and the nanoparticles washed twice with, in sequence, water (2 × 4 mL), EtOH (2 × 4 mL) and DCM (2 × 4 mL) sequentially. Each washing step consisted of suspending the particles in the solvent, sonication (5 min) and retracting the particles from the solvent by the aid of the magnet. After the last washing step, the particles were dried in vacuum overnight and recovered as a black solid of 111 mg. IR (KBr, cm^−1^) *ν* 3435, 2919, 2851, 1620, 1430, 1261, 1095. **CoNPs-A94 (4)**. Turbobeads Click (100 mg, 0.1 mmol/g azide-loading, 0.01 mmol) were washed with degassed toluene (3 × 1 mL) and suspended in the same solvent (4 mL) by sonication (10 min) before **2** (34 mg, 0.1 mmol,) TEA (7 *μ*L, 0.05 mmol) and CuI (1 mg, 0.005 mmol) were added. The resulting slurry was sonicated for 24 h at room temperature under a nitrogen atmosphere, then a second crop of CuI (1 mg, 0.005 mmol) was added and the mixture sonicated for additional 12 h. The nanoparticles were recovered from the reaction mixture with the aid of a neodymium-based magnet and washed with toluene (2 × 9 mL). Adventitious trace of residual Cu(I) salts were removed by washing with a solution of 33% ammonia/EtOH (2/1, 5 mL) under sonication for 30 min, then the solution was removed and the nanoparticles washed twice with, in sequence, water (2 × 4 mL), EtOH (2 × 4 mL) and DCM (2 × 4 mL) sequentially. Each washing step consisted of suspending the particles in the solvent, sonication (5 min) and retracting the particles from the solvent by the aid of the magnet. After the last washing step, the particles were dried in vacuum overnight and recovered as a black solid of 98 mg. IR (KBr, cm^−1^) *ν* 3400, 2952, 2919, 1601, 1457, 1261, 1099.

## Supplementary information

Electronic Supplementary Material

## Data Availability

Electronic Supplementary Material: Further details of autoxidation experiments, semi-quantitative determination of the peroxides, NMR spectra are available in the online version of this article at www.nature.com/articles/s41598-019-53531-5.
