# Supplementary material for: Magnetic nanoantioxidants with improved radical-trapping stoichiometry as stabilizers for inhibition of peroxide formation in ethereal solvents
Source: Sci Rep. 2019 Nov 20;9:17219. doi: 10.1038/s41598-019-53531-5 (PMC6868006; doi:10.1038/s41598-019-53531-5)

Electronic Supplementary Material

| **Magnetic nanoantioxidants with improved radical-trapping stoichiometry as stabilizers for inhibition of peroxide formation in ethereal solvents** |
| --- |
| Caterina Viglianisi^1,*^  Alessia Scarlini^1^, Lorenzo Tofani^1^, Stefano Menichetti^1,^, Andrea Baschieri^2^, and Riccardo Amorati^2,^  *^1^ Department of Chemistry "U. Schiff", University of Florence, Via della Lastruccia 3-13, 50019, Sesto Fiorentino, Firenze, Italy*  *^2^ Department of Chemistry “G. Ciamician”, University of Bologna, Via S. Giacomo 11, 40126, Bologna, Italy.*  Supporting information to DOI 10.1007/s12274-****-****-* (automatically inserted by the publisher) |

1 Determination of Peroxide in solvents - Stability tests on THF

**Materials** The semi-quantitative determination of the peroxides formation in THF was done using commercially available test strips with the trademark QUANTOFIX Peroxide 100.

Quantofix peroxides test sticks, 1-100 mg/L consists of a plastic strip with attached sealed test paper. In the presence of hydroperoxide in concentration between 1 and 100 mg/L, the color of these strips turns from white to blue and a semi-quantification can be done by comparison to a color scale provided on plastic box. The evaluation of the hydroperoxides development in test solutions of THF containing the CoNPs-AntiOx was done using the nanostructured antioxidants sample at 250 ppm. As references were also used a THF sample without stabilizers, the sample with the molecular antioxidant BHT 250 ppm, corresponding to the concentration of the stabilizer in the commercial products. Each THF sample was stored in a 25 mL clear glass bottle closed with a screw top and exposed to light in order to speed up the autooxidation of THF. Because the nanostructured antioxidants were not well dispersed in the solvent, the samples were stirred with a magnetic stir bar in order to facilitate the contact with the hydroperoxides in solution see Figure S1.


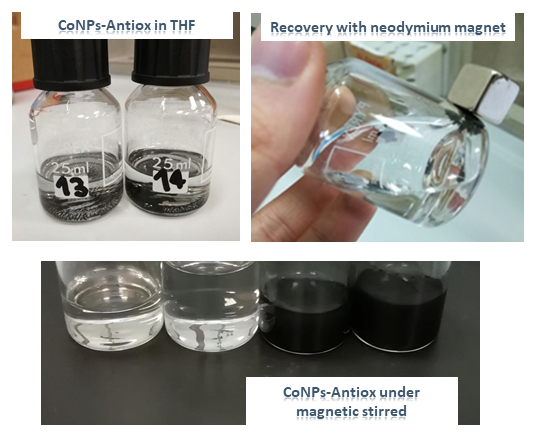
For determining the amount (in weigh) of CoNPs-Antiox to add in order to obtain a content of antioxidant moiety of 250 ppm a simplification was done. We considered that the yield of the functionalization was 100% then, because the functional loading of the commercial nanoparticles is known (0.1 mmol/g), it was possible to calculate the corresponding amount of antioxidant moieties (mmol). In Table 1 is reported the composition of each bottle used for the test.

**Figure S1** CoNPs-Antiox samples suspended in THF and recovered with a neodymium magnet.

**Table S1** Composition of THF samples used for the test stability with antioxidant functionalized Co-NPs.

| **Entry** | **Stabilizer** | **Amount (mg)** | **mmol** | **THF (g)** | **ppm** |
| --- | --- | --- | --- | --- | --- |
| **1** | - | - | - | 10 | - |
| **2** | BHT | 2.5 | 11.5x10^-3^ | 10 | 250 |
| **3** | Turbobeads Click | 115 | 11.5x10^-3^ | 10 | 250 |
| **5** | CoNPs-A80 (**3**) | 111 | 11.5x10^-3^ | 10 | 250 |
| **5** | CoNPs-A94 (**4**) | 111 | 11.5x10^-3^ | 10 | 250 |

**Method of application**: 1. Put a drop of solution tested over the attached sealed test paper of the test strip. 2. Shake off excess liquid. 3. Allow solvent to dry completely. 4. Once dry, moisten the test field with a drop of water. 5. Wait 15 seconds. 6. Compare with the color scale. If hydrogen peroxide is present, the test field turns blue.

As reported in Figure S2 the result of the test sticks indicate that after six days for the solution containing the THF sample without stabilizers the stick turn dark blue corresponding to a concentration of 100 mg/L of peroxides, the sample with the molecular antioxidant BHT 250 ppm have a pale blue green color corresponding at 1 mg/L of peroxide, instead with our great pleasure for the new magnetic stabilizer systems **3** CoNPs-A80 and **4** CoNPs-A94, we have not peroxide presence as demonstrate the white color of the strips.

**
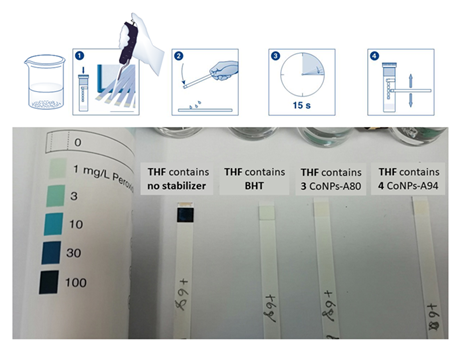

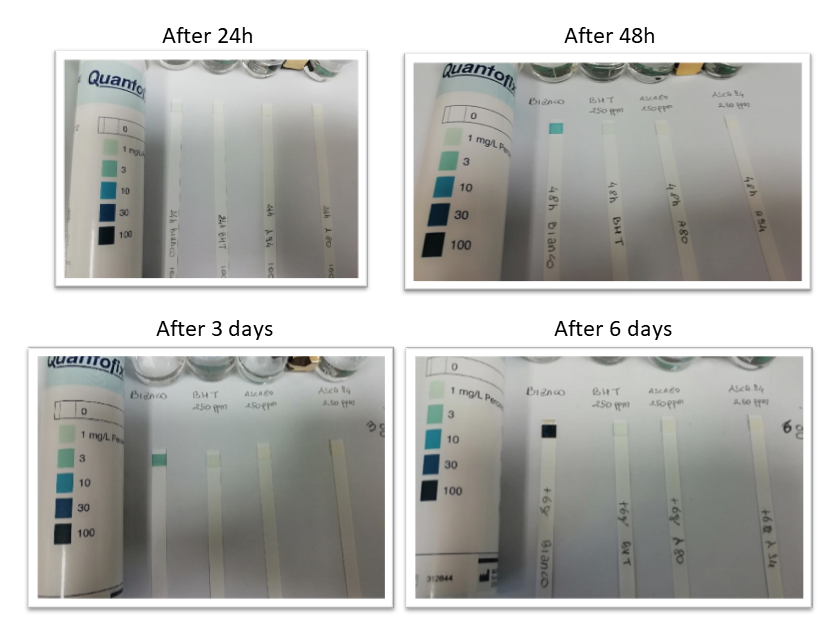
**

**Figure S3** Semi-quantitative determination of hydrogen peroxide and peroxides in THF solutions with results of test strips at different times: after 24 h, 48 h, 3 days and 6 days.

**Figure S2** Schematic rappresentation of Quantofix peroxides test sticks utilization.

2 Measure of the antioxidant activity

**Materials.** Cumene was twice percolated on an alumina column before use. AIBN was recrystallized from methanol. Benzonitrile was washed with diluted NaOH (0.1 M) to remove traces of acids and was dried with anhydrous Na_2_SO_4_.


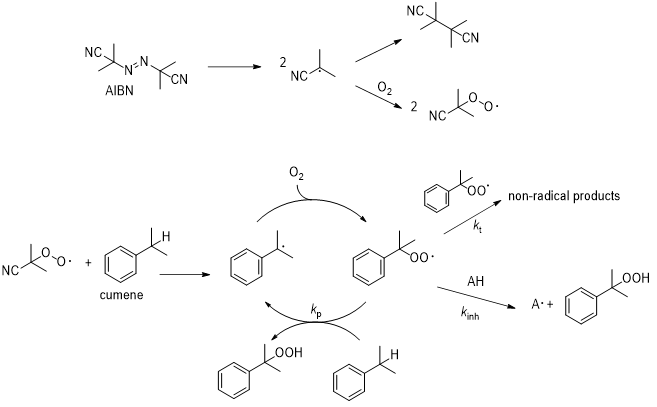
**Autoxidation Experiments.** The antioxidant activity was assessed by measuring the inhibition of the autoxidation of cumene (isopropylbenzene) initiated by AIBN, as reported in Scheme S1.

**Scheme S1.** Mechanism of autoxidation of cumene initiated by AIBN and inhibited by a generic chain-breaking antioxidant AH.

Autoxidation experiments were performed in a two-channel oxygen uptake apparatus, based on a Validyne DP 15 differential pressure transducer built in our laboratory. In a typical experiment, an air-saturated solution of cumene containing AIBN and the investigated nano-antioxidant was equilibrated with an identical reference solution containing excess 2,2,5,7,8-pentamethyl-6-hydroxychromane (PMHC) (25 mM). The oxygen consumption in the sample was measured, after calibration of the apparatus, from the differential pressure recorded with time between the two channels. Initiation rates, *R*_i_, were determined in preliminary experiments by the inhibitor method using PMHC as a reference antioxidant: *R*_i_ = 2[PMHC]/τ, where τ is the length of the induction period (*R*_i_=(2.2±0.1)×10^-9^ M^-1^s^-1^). The inhibition rate constants were determined by using the kinetic equation 1, where d[O_2_]_0_/dt and d[O_2_]_inh_/dt represent the O_2_ consumption in the absence and in the presence of antioxidant, respectively, 2*k*_t_ us the termination constant of cumene (4.6x10^4^ M^-1^s^-1^) and *n* is the stoichiometric coefficient of the antioxidant, determined from the length of the inhibition period by equation 2.


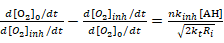


$\frac{{d[O_{2}]}_{0}/dt}{{d[O_{2}]}_{\mathrm{inh}}/dt}-\frac{{d[O_{2}]}_{\mathrm{inh}}/dt}{{d[O_{2}]}_{0}/dt}=\frac{nk_{\mathrm{inh}}[AH]}{\sqrt{2k_{t}R_{i}}}$Equation 1


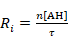


Equation$R_{i}=\frac{n[AH]}{\tau}$ 2

Amorati, R.; Baschieri, A.; Valgimigli, L. Measuring Antioxidant Activity in Bioorganic Samples by the Differential Oxygen Uptake Apparatus: Recent Advances. *J. Chem.* 2017, 2017, 1.


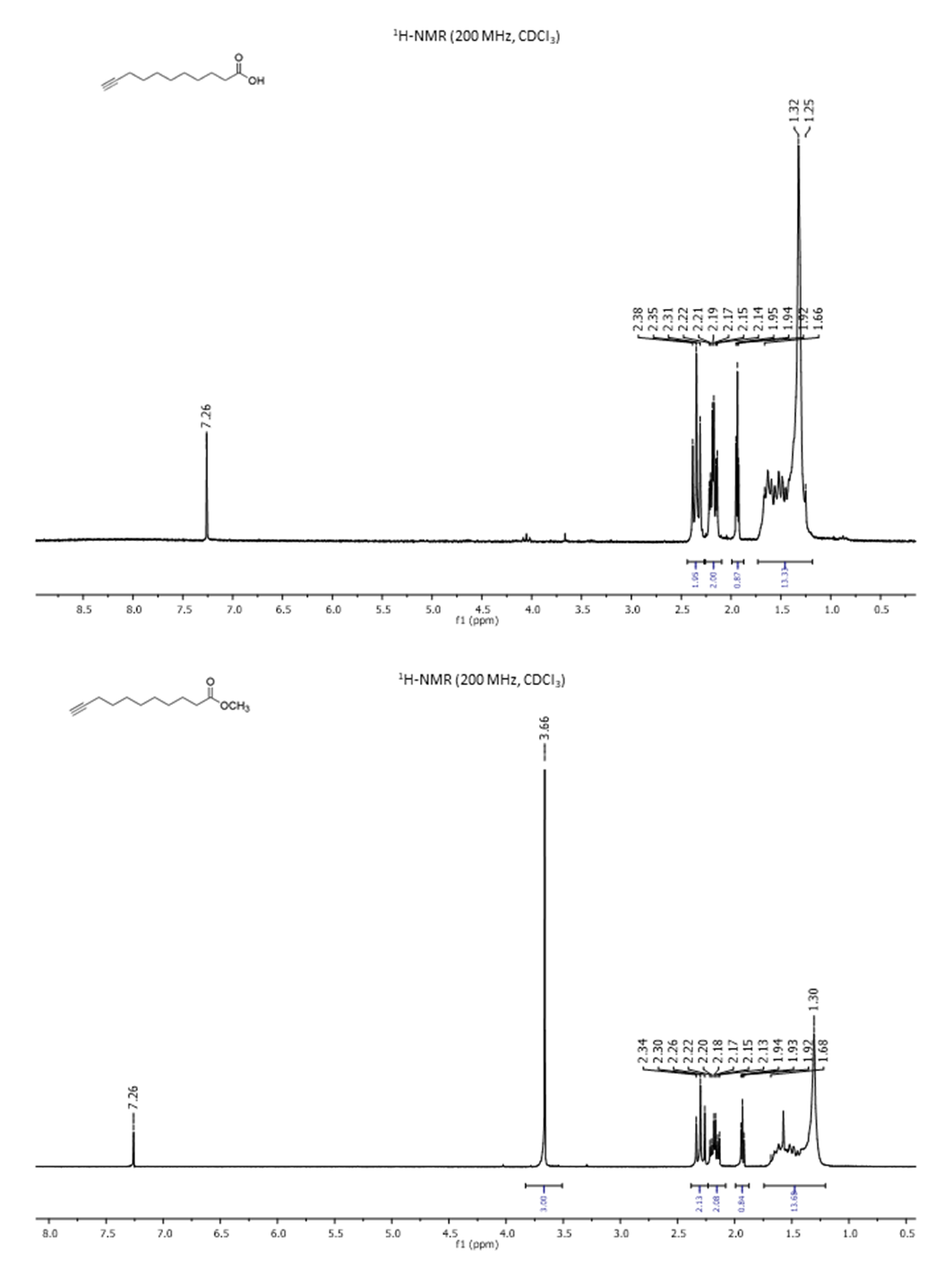
3 NMR Spectra of New Compounds


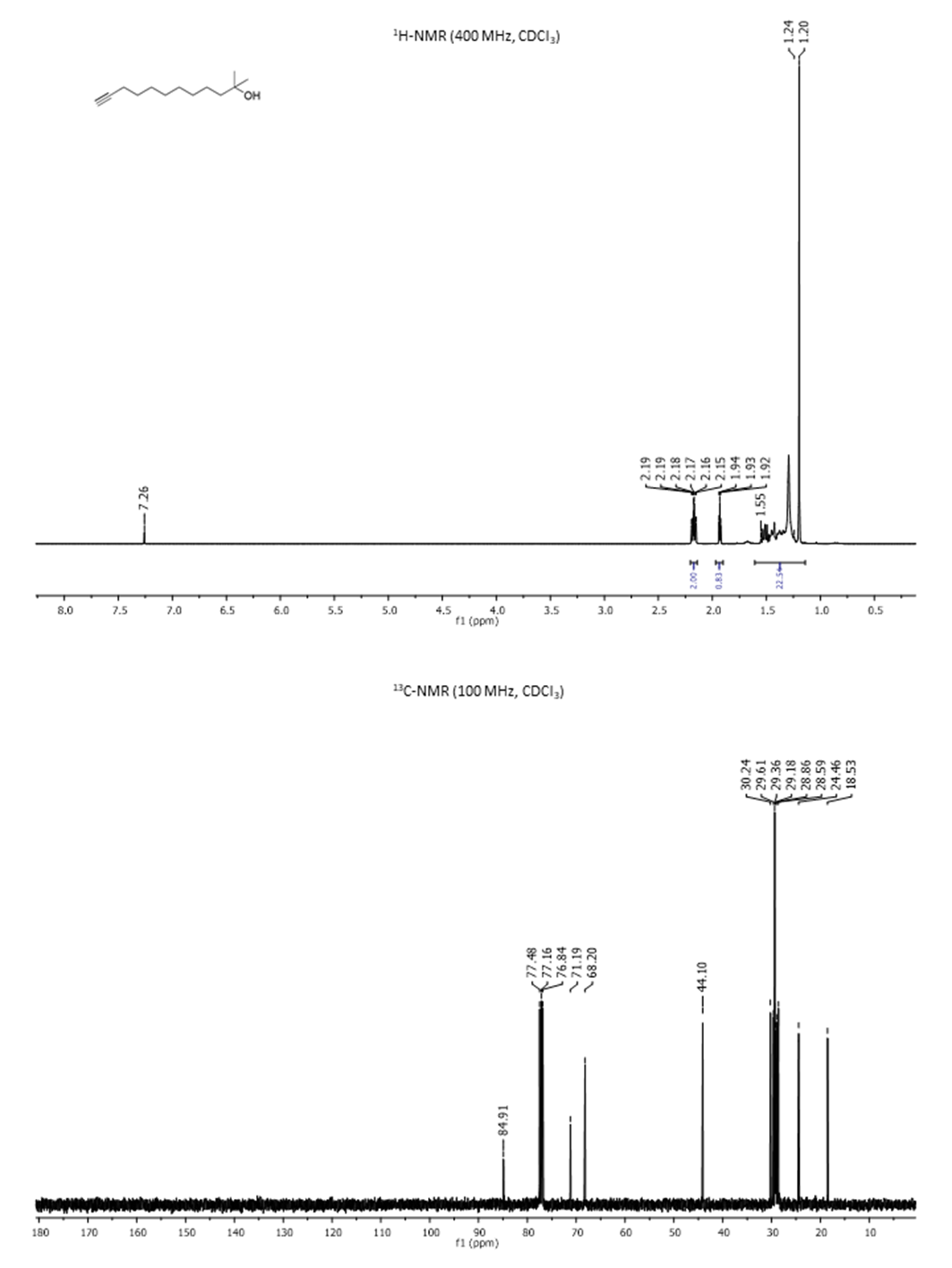


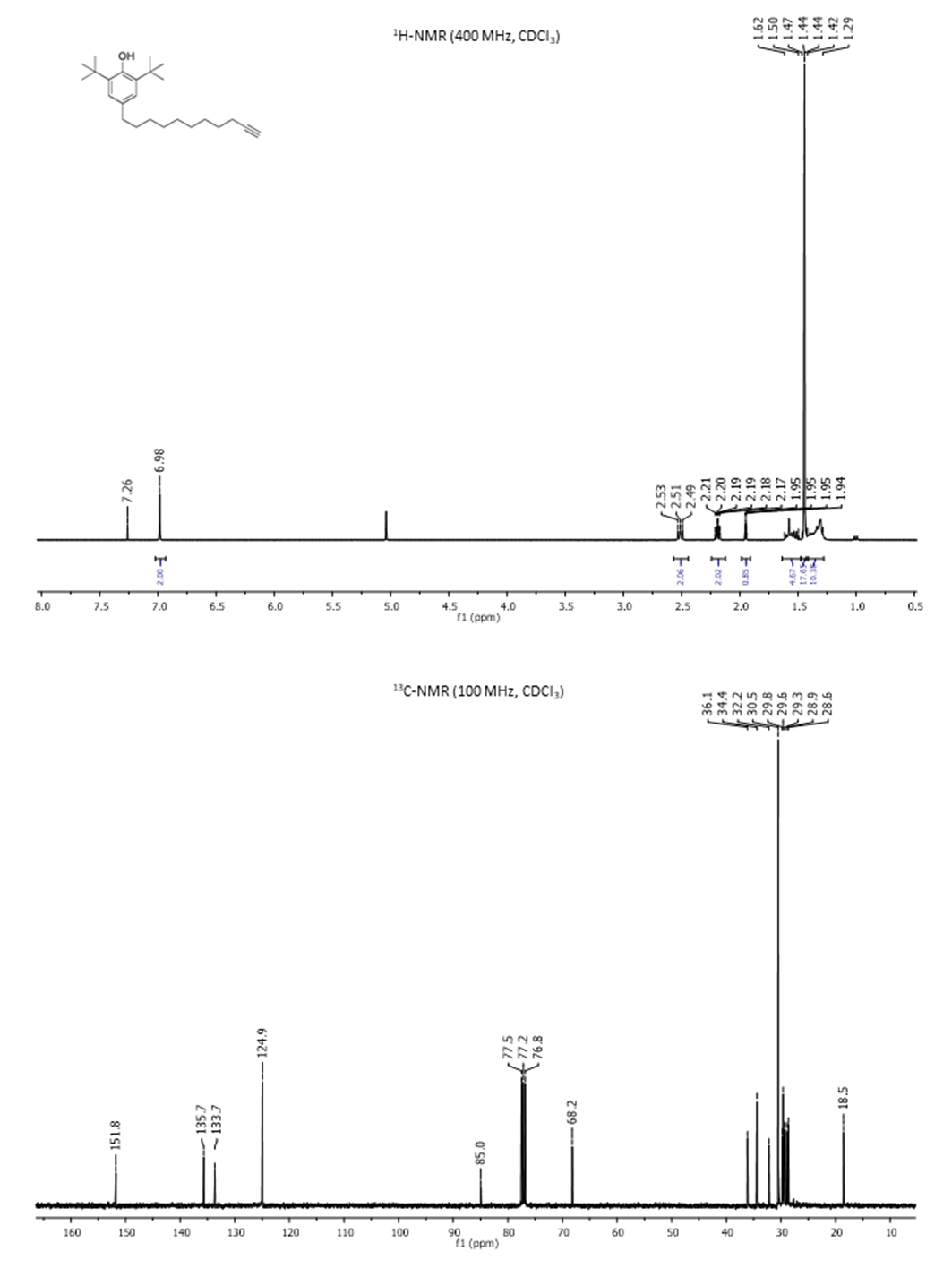


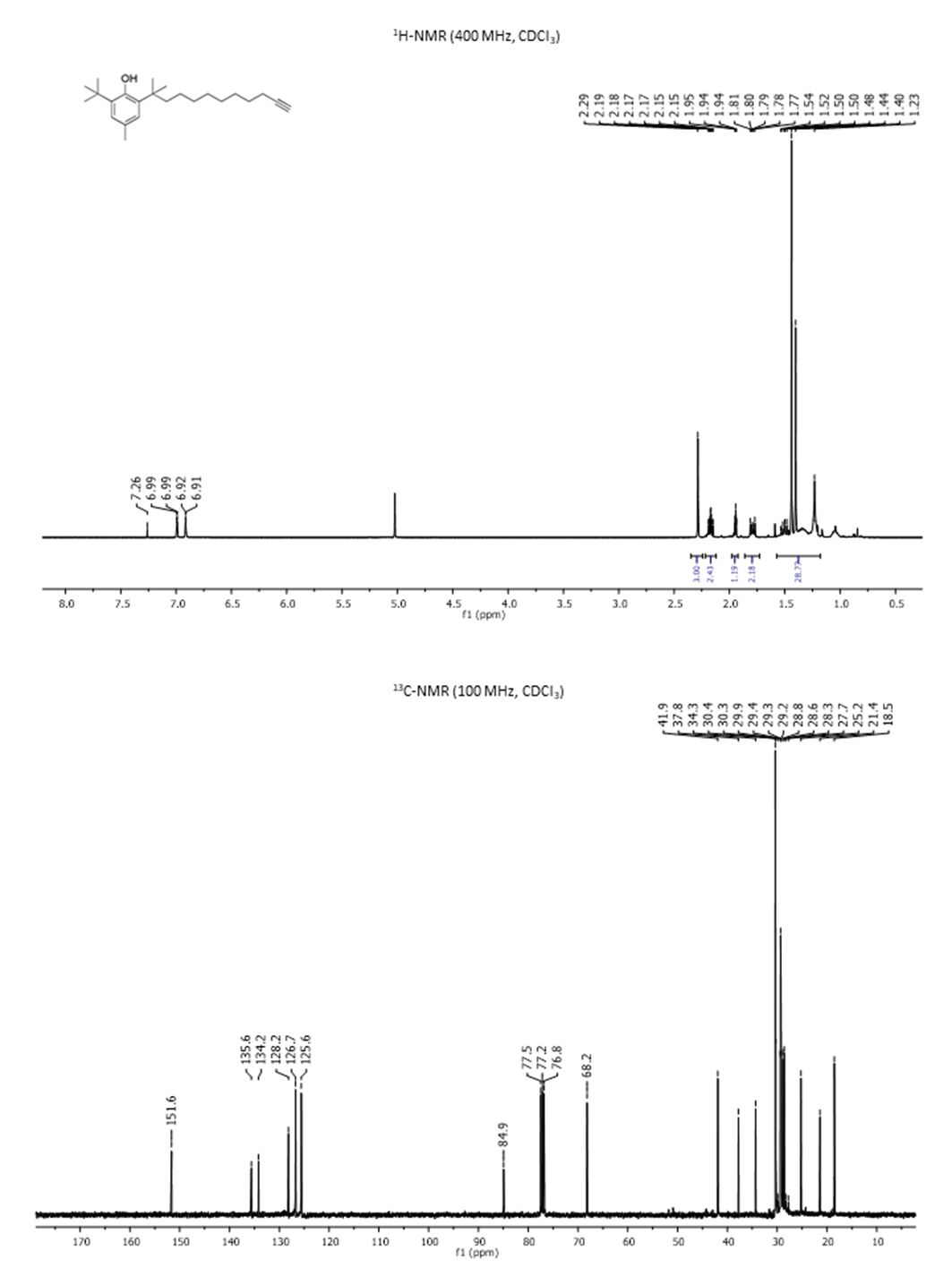

Supplement: Supplementary file 1 — Electronic Supplementary Material [file 41598_2019_53531_MOESM1_ESM.docx]
